# Limitations on knowledge of autoimmune encephalitis and barriers to its treatment among neurologists: a survey from western China

**DOI:** 10.1186/s12883-023-03139-0

**Published:** 2023-03-07

**Authors:** Aiqing Li, Kundian Guo, Xu Liu, Xue Gong, Xingjie Li, Dong Zhou, Zhen Hong

**Affiliations:** 1 Department of Neurology, West China Hospital, Sichuan University, No. 37 Guoxue Road, Chengdu, Sichuan 610041 China; 2 Department of Neurology, Chengdu Shangjin Nanfu Hospital, Chengdu, Sichuan 611730 China; 3 Institute of Brain Science and Brain-inspired Technology, West China Hospital, Sichuan University, Chengdu, Sichuan 610041 China

**Keywords:** Autoimmune encephalitis, Knowledge, Practice, Perspective, Western China

## Abstract

**Background:**

Autoimmune encephalitis (AE) is a group of severe antibody-mediated brain diseases. The understanding of clinical management of AE has developed rapidly. However, the knowledge level of AE and barriers to effective treatment among neurologists remains unstudied.

**Methods:**

We conducted a questionnaire survey among neurologist in western China on knowledge of AE, treatment practices, and perspectives on barriers to treatment.

**Results:**

A total of 1113 neurologists were invited and 690 neurologists from 103 hospitals completed the questionnaire with a response rate of 61.9%. Respondents correctly answered 68.3% of medical questions about AE. Some respondents (12.4%) never assayed for diagnostic antibodies if patients had suspected AE. Half (52.3%) never prescribed immunosuppressants for AE patients, while another 7.6% did not know whether they should do so. Neurologists who never prescribed immunosuppressants were more likely to have less education, a less senior job title, and to practice in a smaller setting. Neurologists who did not know whether to prescribe immunosuppressants were associated with less AE knowledge. The most frequent barrier to treatment, according to respondents, was financial cost. Other barriers to treatment included patient refusal, insufficient AE knowledge, lack of access to AE guidelines, drugs or diagnostic test, etc.

**Conclusion:**

Neurologists in western China lack AE knowledge. Medical education around AE is urgent needed and should be more targeted to individuals with less educated level or working in non-academic hospitals. Policies should be developed to increase the availability of AE related antibody testing or drugs and reduce the economic burden of disease.

**Supplementary Information:**

The online version contains supplementary material available at 10.1186/s12883-023-03139-0.

## Introduction

Autoimmune encephalitis (AE) is a group of inflammatory brain diseases that are primarily associated with antibodies against neuronal cell-surface proteins, ion channels, or receptors, and is manifested by memory dysfunction, psychiatric symptoms, involuntary movement, autonomic dysfunction, reduced level of consciousness, and seizures [1]. The most common AE subtypes involve antibodies against N-methyl-d-aspartate receptor (NMDAR), leucine-rich glioma-inactivated 1 (LGI1) protein, contactin-associated protein-like 2 (Caspr2) protein, or γ-aminobutyric acid type B receptor (GABA_B_R) [1]. Since anti-NMDAR encephalitis were first discovered in 2007, AE have become new research hotspot in the field of neurological diseases over the past decade. The understanding of various aspects of AE such as pathogenesis, clinical features, and clinical management has developed rapidly [1]. The first consensus guidelines for diagnosing the disease were published in the US in 2016 [1], and China published national consensus guidelines on clinical management of AE in 2017 [2]. The first International Consensus Recommendations for the treatment of pediatric NMDAR encephalitis have just been released in 2021 [3]. AE is as prevalent as infectious encephalitis [4]. It is estimated that the incidence rates (1995–2015) of autoimmune and infectious encephalitis were 0.8/100,000 and 1.0/100,000 person-years, respectively (p = 0.58) [4]. Antibody testing is critical for the diagnosis of AE [1]. Previous studies [5–7], including our own work, suggest that 70–80% of patients with AE are highly responsive to first-line immunotherapy (high-dose intravenous methylprednisolone (IVMP), intravenous immunoglobulin (IVIG) and plasma exchange). Second-line IT (IT), including immunosuppressants such as cyclophosphamide and rituximab were suggested if there is no meaningful clinical or radiological response to first-line IT [5–7]. Some AE patients require long-term hospitalization and intensive care, which place a heavy economic burden on families and society. However, patients with AE in certain ethnic groups or in certain countries may lack access to effective treatments, perhaps because they are unavailable or neurologists are unaware of them [6, 8–11]. This situation is hardly surprising, given that knowledge about AE and its treatment is relatively new. The rapid advances of AE research in the past 15 years necessitates commensurate endeavors among neurologists to keep up with the new knowledge.

The importance of adequate disease knowledge and access to tests or therapies is clear from studies of epilepsy, Alzheimer’s disease and Parkinson’s disease, particularly in developing nations [12–14]. These surveys reported that the recommended diagnostic tests are unavailable, which limited the opportunity for prompt recognition and treatment of diseases [12–14]. For some diseases, effective treatments may simply be unavailable, or clinicians and their patients may balk at their high cost, potential adverse effects, or off-label use [15, 16]. While, so far, the knowledge level of AE and barriers to effective treatment practices among neurologists remains unstudied.

China is a vast, diverse, a developing country with rapid development [17, 18]. It is of great significance for health decision-making in China and even the world to deeply understand the current understanding and practice of AE by Chinese neurologists. Compared with the eastern region of China, the resources in the western China are more deficient [17, 18]. Here, we chose to focus on western China because poverty and lack of medical resources represent particular challenges to ensuring universal access to suitable care in the region for patients with AE [18].

For the first time, we conducted a cross-sectional online survey of neurologists at several healthcare organizations in western China to assess their AE knowledge as well as understand their treatment practices and perspectives on barriers to effective treatment.

## Methods

### Participants and survey

The survey was initiated and directed by the Sichuan Medical Doctor Association (SMDA) from May 22, 2021 to November 22, 2021. SMDA is a society composed of medical experts active in clinical care and research at 177 medical centers in western China, which aims to promote clinical management as well as scientific research, academic exchange, and dissemination of scientific knowledge of medicine. We invited 35 neurologists who were current members of the Nervous System Infection and Cerebrospinal Fluid Study Group (NICG) of the SMDA to participate. Members of this Study Group focus on treating AE patients and researching the disease. We also invited 1078 neurologists from 103 hospitals in western China who were not members of this Study Group. All the participants we invited to participate were validated as neurologists by SMDA and provided contact information. To be considered eligible for this study, neurologists had to be currently working in a neurology department, and they had to be the registered WeChat users. All participants were invited to complete the questionnaire on-line via WeChat. WeChat is a simple Chinese mobile applet, like WhatsApp in some countries, widely used and convenient. The WeChat-based approach has been widely applied in questionnaire collection studies in China [19]. No monetary or other compensation was offered to the participants. Respondents had to provide informed consent before the survey began, and they were told that their responses would be anonymous. This study was approved by the Research Ethics Committee of West China Medical Center of Sichuan University.

Neurologists were excluded if they submitted a survey twice from the same IP address, if they completed the questionnaire in less than three minutes, or if they had participated in the testing of the content validity and test-retest reliability of the knowledge section of the questionnaire related to this survey. This study followed the relevant portions of the Strengthening the Reporting of Observational Studies in Epidemiology (STROBE) reporting guideline for cross-sectional analysis and the American Association for Public Opinion Research (AAPOR) reporting guideline for survey studies.

### Questionnaire

Using the survey tool “So Jump” [20], three authors (X.L, Z.D, and Z.H) drafted a questionnaire (see eAppendix in the Supplement) based on their own extensive experience treating AE and researching the disease within numerous published projects [5–7, 21–23]. They aligned the questionnaire content to the Chinese Expert Consensus on AE Management [2]. The content validity and test-retest reliability of the knowledge section of this questionnaire were assessed (see eTable1 and eMethods in the Supplement) [24]. The correlation coefficients were 1.0 for content validity and 0.9 for test-retest reliability. The questionnaire contained three parts (Part A, Part B and Part C). More detailed description of the questionnaire can be seen in the eMethods in the supplement. Additional description of terms and definitions used on the survey are explained as follow [25]:

House physician: primary title, practice under the guidance and supervision of the attending physician or above.

Attending physician: middle job title, higher than the house physician, lower than the assistant director physician.

Assistant director physician: vice-senior title, equivalent to associate professor, higher than the attending physician, lower than the director physician.

Director physician: senior title, equivalent to professor.

In China, all hospitals are classified into 3 levels: primary, secondary, and tertiary [26]. Primary hospitals (primary level, < 100 beds normally) aim to provide basic public health services and consulting for their residents. Secondary (moderate level, 101–500 beds normally) and tertiary (high level, ≥ 500 beds normally) hospitals provide specialized care. In addition, the tertiary hospitals could be divided to academic tertiary hospitals and non-academic tertiary hospitals. Clinicians working in academic tertiary hospitals have duty of conducting clinical/basic research, teaching and tutoring in addition to daily clinical work.

### Statistical analysis

Responses were stratified according to the following variables and categories: (1) *age in years*, 22–30, 31–45, or 46–65; (2) *years practicing neurology*, < 10, 11–20, > 20; (3) *education level*, bachelor’s degree, master’s degree, or doctorate; (4) *practice setting*, academic tertiary hospital, non-academic tertiary hospital, or secondary hospital; (5) *job title*, house physician, attending physician, assistant director physician, or director physician; (6) *membership in NICG*, yes or no; and (7) *AE knowledge level*, ≥ the median or below the median.

We summarized descriptive statistics and compared subgroups using the χ^2^ test for categorical variables or the *t* test for continuous variables. Statistical analyses were performed in SPSS Statistics for Windows (version 20.0; IBM, Armonk, NY, USA). Statistical significance was defined as two-sided p < 0.05.

## Results

### Demographic features of the participants

**Fig. 1:**
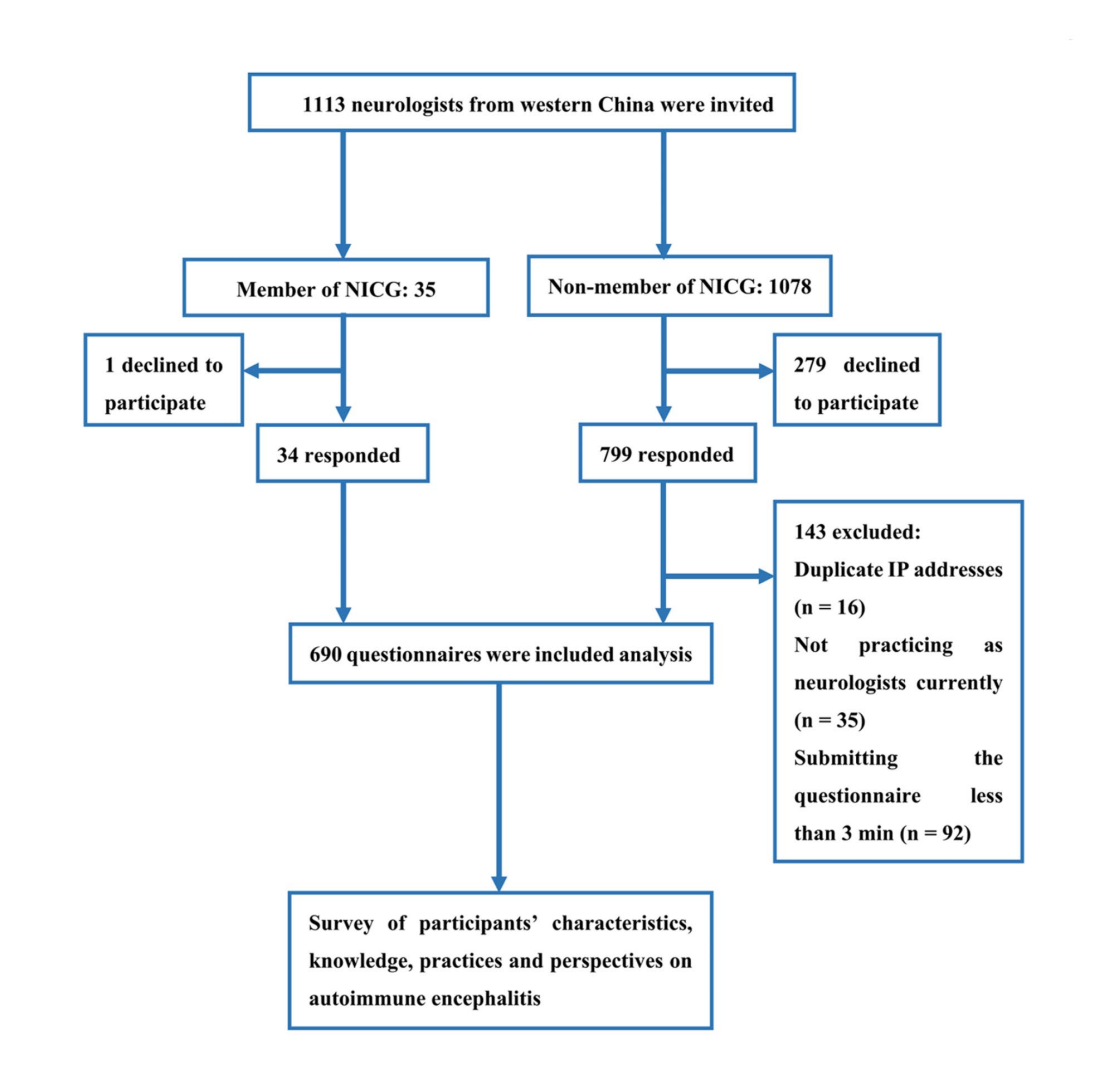
The flowchart of the participants in the survey NICG, Nervous System Infection and Cerebrospinal Fluid Study Group

**Fig. 2:**
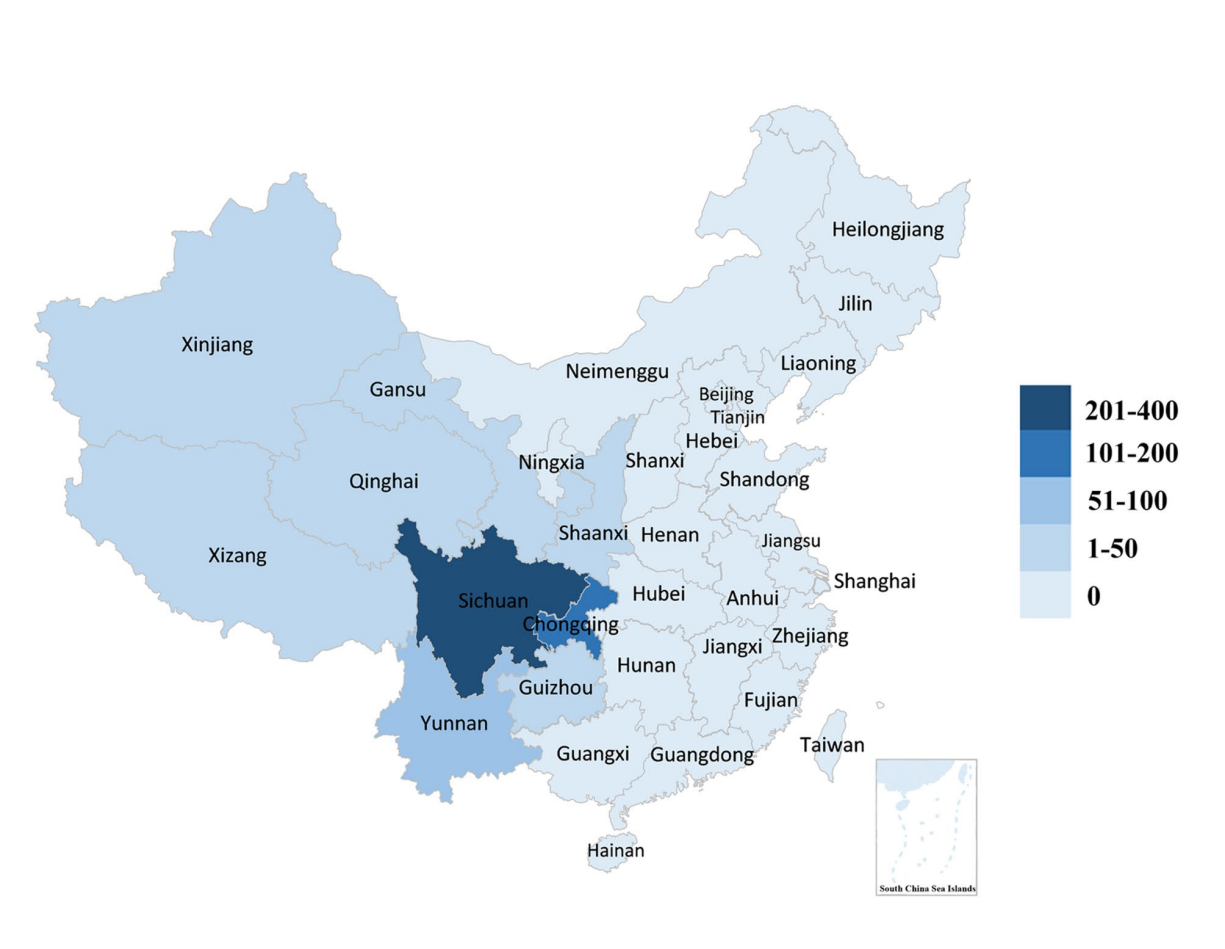
The distribution of respondents in western provinces of China The numbers of respondents in 9 western provinces of China are showed

The flowchart of the participants is showed in Fig. 1. A total of 1113 neurologists were invited, 833 responded to the survey, 143 were excluded (16 of them had duplicate IP addresses, 35 of them did not practice as neurologists at the time of survey and the rest of them submitted the questionnaire less than 3 min). The final analysis involved 690 neurologists from 103 hospitals. The distribution of respondents in western provinces of China is shown in the Fig. 2. Participants came from 9 provinces in western China, of which 53.0% were from Sichuan Province, and at least 10 qualified respondents from each province participated.

The demographic and clinical features of the participants are show in Table 1. Compared to respondents who were non-members of the NICG, respondents who were members of the NICG were older, had a higher education level, more senior job title, had been practicing neurology longer, had contact with more cases of encephalitis and AE annually (p < 0.001).

**Table 1:** Sociodemographic and clinical characteristics of respondents

Characteristic	NICG membership		Total (N = 690)	P
	Yes (n = 34)	No (n = 656)		
Sex				0.73
Female	22 (64.7)	424 (64.6)	446 (64.6)	
Age, yr				< 0.001
22–30	0 (0.0)	253 (38.6)	253 (36.7)	
31–45	27 (79.4)	370 (56.3)	397 (57.5)	
46–65	7 (20.6)	33 (5.1)	40 (5.8)	
Years practicing neurology				< 0.001
≤ 10	5 (14.7)	487 (74.3)	492 (71.3)	
11–20	22 (64.7)	131 (19.9)	153 (22.2)	
> 20	7 (20.6)	38 (5.8)	45 (6.5)	
Education level				< 0.001
Bachelor’s degree	5 (14.7)	270 (41.2)	275 (39.9)	
Master’s degree	18 (52.9)	337 (51.4)	355 (51.4)	
Doctorate	11 (32.4)	49 (7.4)	60 (8.7)	
Practice setting				0.799
Academic tertiary hospital	14 (41.2)	234 (35.7)	248 (35.9)	
Non-academic tertiary hospital	20 (58.8)	407 (62.1)	427 (61.9)	
Secondary hospital	0 (0.0)	15 (2.3)	15 (2.2)	
Job title				< 0.001
House physician	0 (0.0)	234 (35.7)	234 (33.9)	
Attending physician	10 (29.4)	296 (44.9)	306 (44.3)	
Assistant director physician	19 (55.9)	70 (10.6)	89 (12.9)	
Director physician	5 (14.7)	26 (3.9)	31 (4.5)	
Number of all-cause encephalitis cases per year				< 0.001
< 10	1 (2.9)	154 (23.5)	155 (22.5)	
10–30	9 (26.5)	295 (45.0)	304 (44.1)	
31–60	13 (38.2)	110 (16.7)	123 (17.8)	
61–90	4 (11.8)	21 (3.2)	25 (3.6)	
> 90	5 (14.7)	26 (3.9)	31 (4.5)	
Unclear	2 (5.9)	50 (7.7)	52 (7.5)	
Number of autoimmune encephalitis cases per year				0.001
< 1	0 (0.0)	36 (5.5)	36 (5.2)	
1–5	9 (26.5)	281 (42.8)	290 (42.0)	
6–10	8 (23.5)	163 (24.8)	171 (24.8)	
11–20	10 (29.4)	66 (10.0)	76 (11.0)	
> 20	6 (17.6)	44 (6.8)	50 (7.2)	
Unclear	1 (2.9)	66 (10.1)	67 (9.7)	

NICG, Nervous System Infection and Cerebrospinal Fluid Study Group

Additional characteristics of the respondents can be seen in the eResults in the Supplement.

### AE knowledge

Table 2 shows the average correct rate for each AE knowledge question. On average, respondents answered 68.3% of knowledge questions correctly. corresponding to a mean KS of 9.6 with a standard deviation of 2.7. On knowledge questions about disease prognosis, respondents answered an average of 61.5% correctly. Respondents answered fewer diagnosis and prognosis questions correctly when the subject was rare AE types (57.9% and 45.2%, respectively) than when it was all AE types (74.2% and 64.0%) or anti-NMDAR encephalitis (88.1% and 75.3%) (P < 0.05). They answered the question about IT correctly far more often than the question about tumor resection (87.8% vs. 65.5%) (P < 0.05).

**Table 2:** Rates of correct responses to questions about autoimmune encephalitis knowledge

Area of knowledge	Question (correct answer) *	Type(s) of AE	Rate of correct responses, %*	Average rate for area of knowledge (%)		
Diagnosis	If a patient is negative for AE-related antibodies, a diagnosis of AE can be ruled out. (F)	All types of AE	91.9	74.2		73.5
	Patients with AE usually have an elevated number of nucleated cells in cerebrospinal fluid. (F)		72.5			
	Many patients with AE show obvious brain abnormalities by magnetic resonance imaging. (F)		58.3			
	The main clinical manifestations of anti-NMDAR encephalitis are psychiatric symptoms, seizures, dyskinesia, and disturbed consciousness. (T)	Anti-NMDAR encephalitis	95.9	88.1		
	Some patients with anti-NMDAR encephalitis show abnormal delta brushes in electroencephalography. (T)		80.3			
	The most frequent tumor among patients with anti-GABA_B_R encephalitis is teratoma. (F)	Rare types of AE	37.1	57.9		
	The main clinical manifestations of anti-IgLON5 encephalitis are parasomnia, sleep disorder, cognitive impairment, and gait abnormality. (T)		74.8			
	Brain magnetic resonance imaging of some patients with anti-GFAP encephalitis shows linear perivascular enhancement that extends radially from the ventricles. (T)		61.8			
Treatment	Immunotherapy is the core treatment for patients with AE. (T)	All types of AE	87.8	76.7		
	Active tumor removal is recommended for patients with both anti-NMDAR encephalitis and teratoma who develop disturbed consciousness. (T)	Anti-NMDAR encephalitis	65.5			
Prognosis	AE usually shows a single course and does not recur. (F)	All types of AE	64.0		61.5	
	Most patients with anti-NMDAR encephalitis have good long-term functional outcomes. (T)	Anti-NMDAR encephalitis	75.3			
	More than 80% of patients with GABA_B_R encephalitis have good long-term prognosis and extremely low risk of mortality. (F)	Rare types of AE	31.3	45.2		
	Patients with anti-LGI1 encephalitis are more likely to show cognitive dysfunction than patients with other types of AE. (T)		59.1			

* Possible responses: “true” (T), “false” (F) or “not sure”

AE = autoimmune encephalitis; NMDAR = anti-N-methyl-d-aspartate receptor; GABABR = anti-γ-aminobutyric acid receptor type B; LGI1 = anti-leucine-rich glioma-inactivated 1; GFAP = anti-glial fibrillary acidic protein

**Fig. 3:**
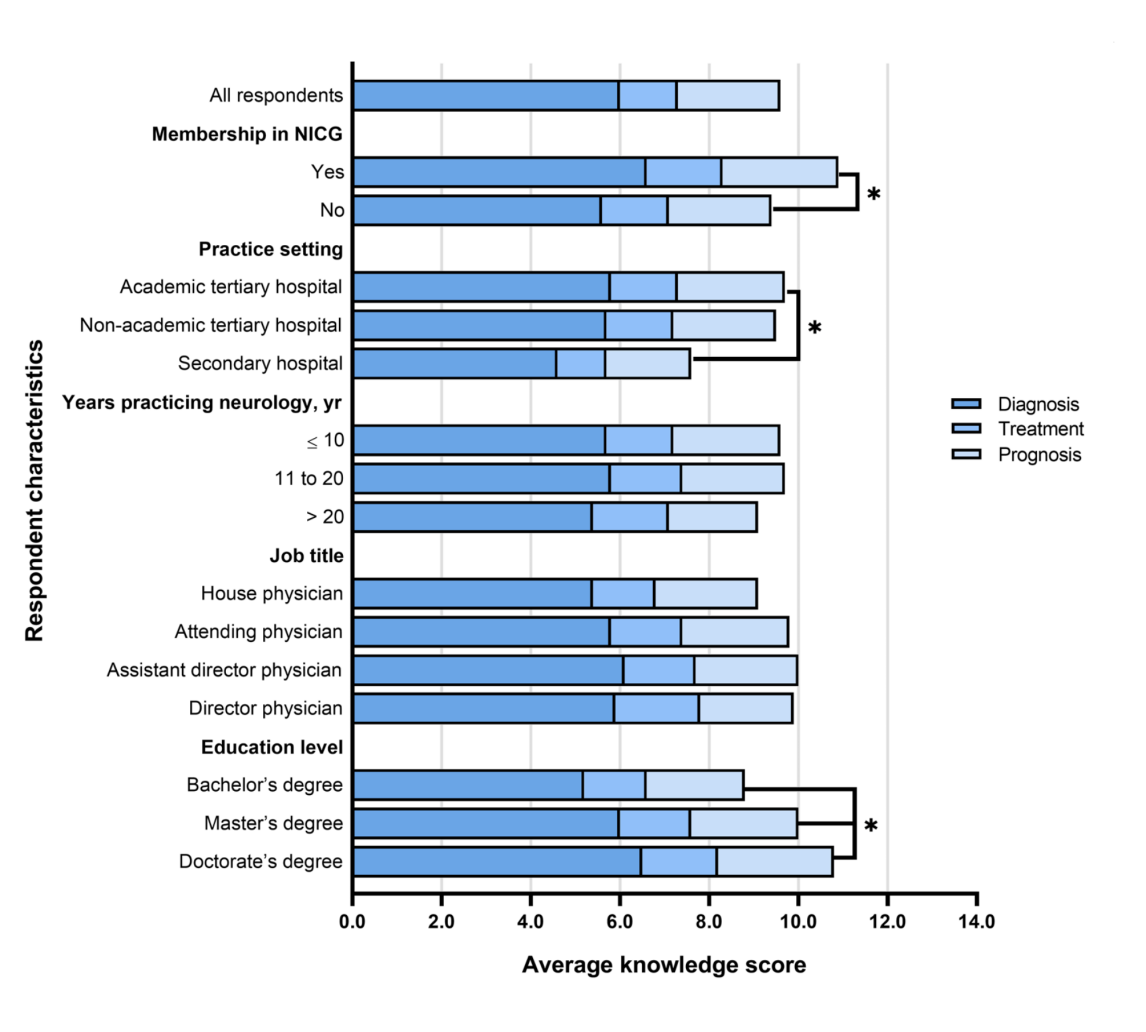
Average knowledge score of autoimmune encephalitis (AE) among neurologists by different characteristic subgroups in western China Results are shown for questions about AE diagnosis, treatment and prognosis. * two-sided p < 0.05 NICG, Nervous System Infection and Cerebrospinal Fluid Study Group

Average KS of AE among neurologists by different characteristic subgroups are showed in Fig. 3. Members of the NICG demonstrated significantly higher mean KS than non-members (10.8 ± 2.3 vs. 9.5 ± 2.8, p = 0.008). Respondents working in academic tertiary hospitals showed significantly higher KS than those working in secondary hospitals (9.8 ± 2.8 vs. 7.6 ± 2.6, p < 0.001). There were significant differences in the KS of respondents with three different education levels: doctorate (10.8 ± 1.7) > master’s degree (10.0 ± 2.6) > bachelor’s degree (8.8 ± 2.8) (p < 0.05). In contrast, AE knowledge level did not seem to vary significantly with job title or years practicing neurology.

### Practices and perspectives on diagnostic antibody testing

Since antibody detection plays an important role for the diagnosis of AE, we investigated the frequency of antibody submission and possible difficulties faced by neurologists in patients with suspected AE. 77.2% of the respondents reported that they always ordered diagnostic antibody testing for patients with suspected AE, while 12.4% never ordered such testing.

**Fig. 4:**
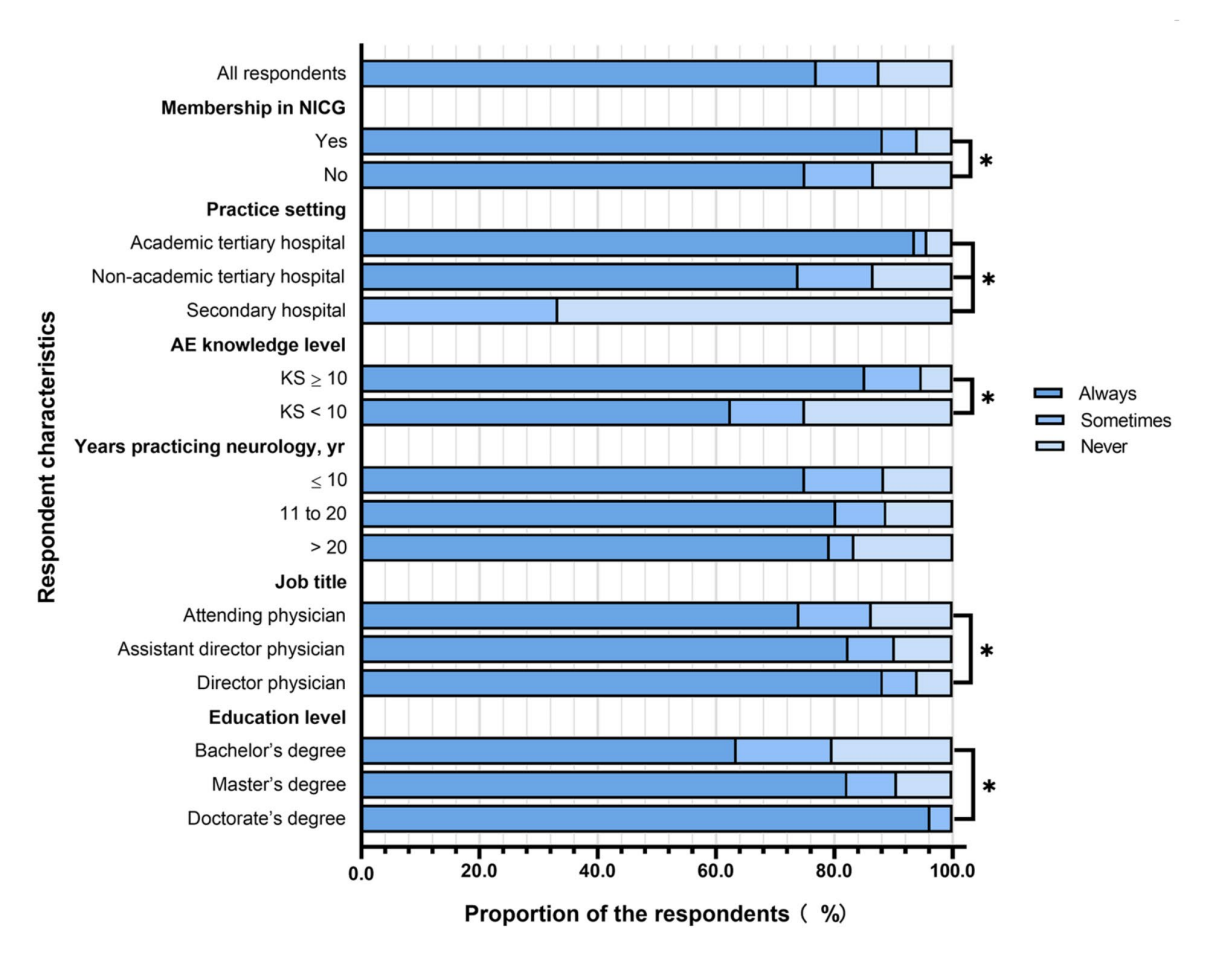
Frequency of ordering antibody testing for patients with suspected autoimmune encephalitis (AE) among neurologists by different characteristic subgroups in western China. Responses to the question, “How often have you ordered diagnostic antibody testing for patients with suspected AE?”. * two-sided p < 0.05 KS, knowledge score; NICG, Nervous System Infection and Cerebrospinal Fluid Study Group

Frequency of ordering antibody testing for patients with suspected AE among neurologists by different characteristic subgroups are also showed in Fig. 4. The proportion of those who never ordered testing was significantly lower among members of the NICG than among non-members (5.9% vs. 13.3%, p < 0.05). Conversely, the proportion of those who never ordered testing was significantly higher among respondents at secondary hospitals (66.7%) than among those at non-academic tertiary hospitals (13.4%) or academic tertiary hospitals (4.3%). The proportion was significantly higher among those with KS < 10 (25.0% vs. 5.2%, p < 0.05), those with a job title of attending physician rather than a more senior title (13.7% vs. 5.9%, p < 0.05), and those with a bachelor’s degree (20.3%) rather than those with a master’s degree (9.3%) or doctorate (0%).

**Fig. 5:**
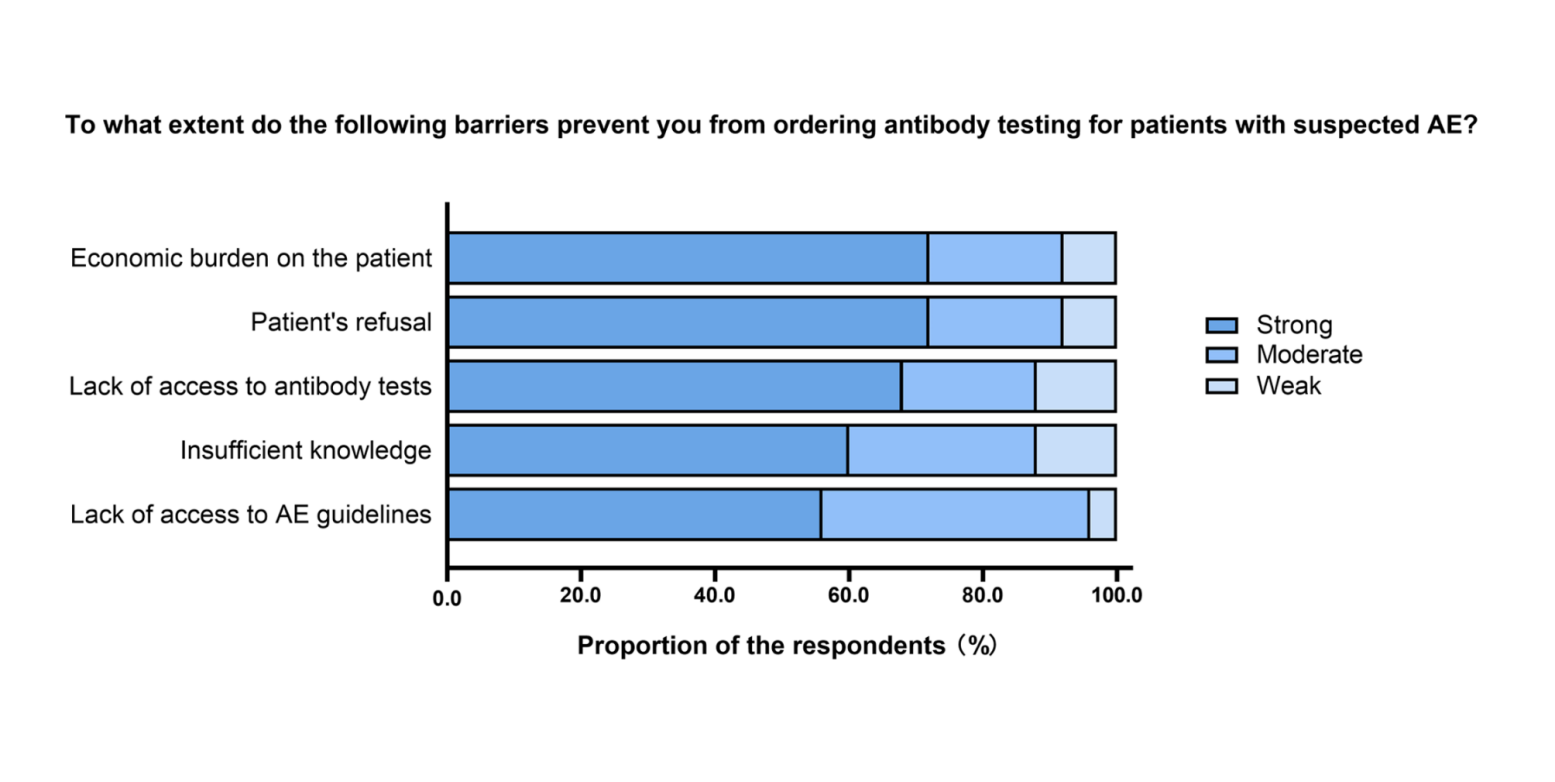
Barriers to ordering antibody testing for patients with suspected autoimmune encephalitis (AE) among neurologists Responses to the question, “To what extent do the following barriers prevent you from ordering diagnostic antibody testing for patients with suspected AE?”

We further investigated the reasons for the interviewees who never tested the patients for antibodies (Fig. 5). “Economic burden on the patient” and “Patient’s preference” were the most common reasons for not submitting antibodies for testing. The following reasons included “Lack of access to antibody tests”, “Insufficient knowledge”, and “Lack of access to AE guidelines”.

### Practices and perspectives on immunosuppressants

As immunosuppressants are one of the important treatment measures for AE, and the use of immunosuppressants may face great challenges due to problems such as exceeding the instructions. Therefore, we further investigated the use of immunosuppressants by Chinese neurologists in AE patients. Over half (52.3%) of the respondents said that they never prescribed immunosuppressants for AE patients, and 7.6% did not know whether they should do so.

**Fig. 6:**
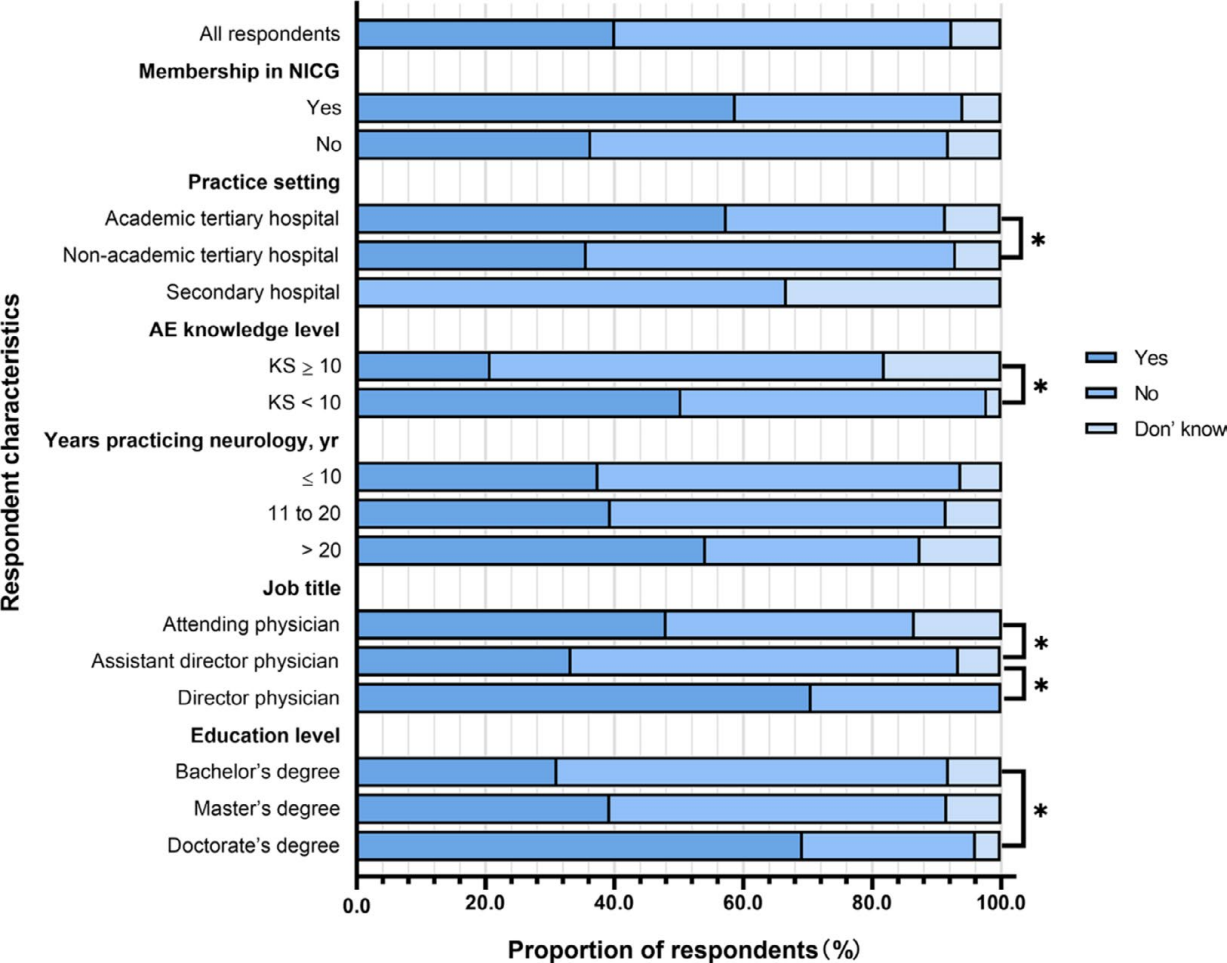
Prescription of immunosuppressants for patients with autoimmune encephalitis (AE) among neurologists by different characteristic subgroups in western China Responses to the question “Have you ever prescribed immunosuppressants for patients with AE?” * two-sided p < 0.05 KS, knowledge score; NICG, Nervous System Infection and Cerebrospinal Fluid Study Group

Prescription of immunosuppressants for patients with AE among neurologists by different characteristic subgroups are also showed in Fig. 6. The proportion of respondents who never prescribed immunosuppressants was significantly higher among those in non-academic tertiary hospitals than among those in academic tertiary hospitals (57.3% vs. 34.0%, p < 0.05), and significantly higher among those with a job title of attending physician (60.1%) than among those with a title of director physician (29.4%) or assistant director physician (38.5%). The proportion was also significantly higher among respondents with a bachelor’s degree than among those with a doctorate (60.8% vs. 26.9%, p < 0.05). The proportion of respondents who did not know whether they should prescribe such treatment was significantly higher among those with KS < 10 (18.1% vs. 2.2%, p < 0.05). Whether respondents prescribed immunosuppressants or knew whether they should do so did not vary significantly with membership in the NICG or with years practicing neurology.

**Fig. 7:**
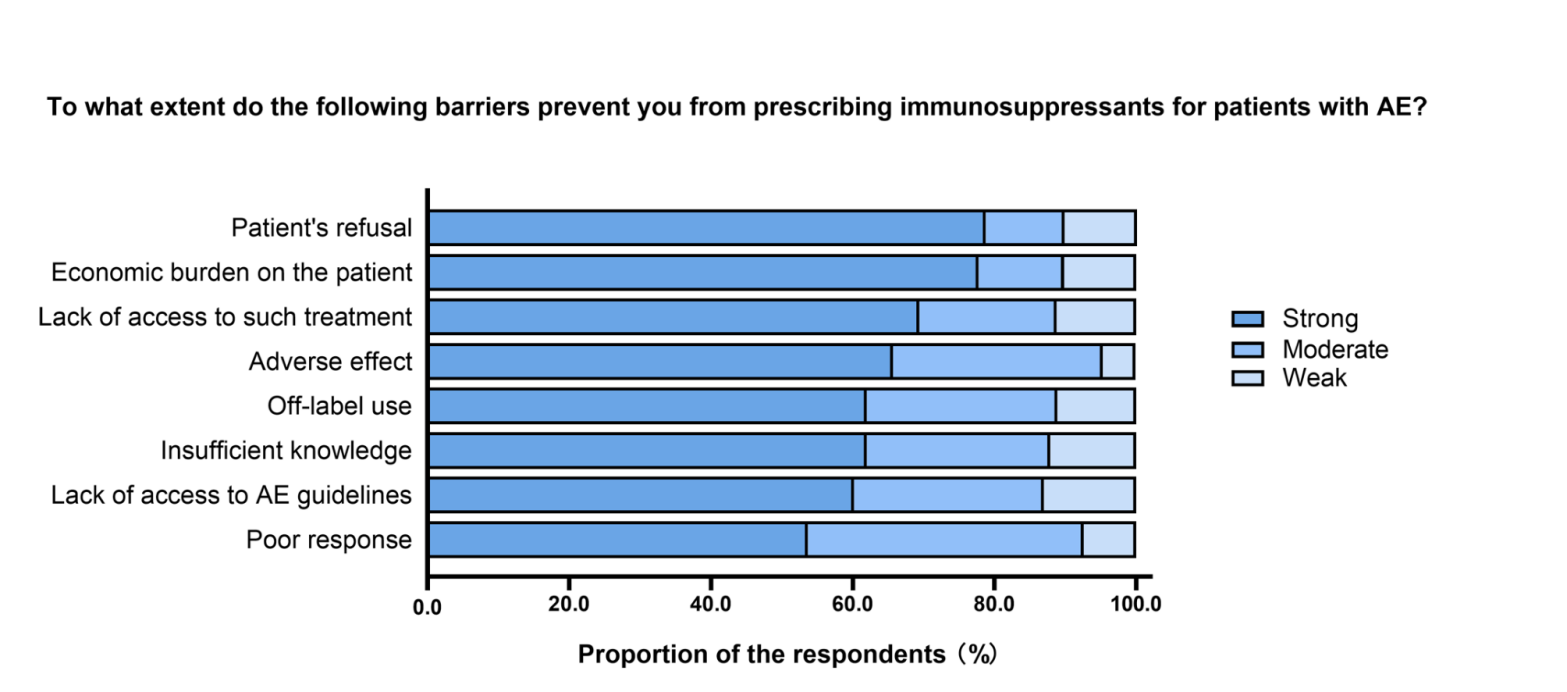
Barriers to prescribing immunosuppressants for patients with autoimmune encephalitis (AE) among neurologists Responses to the question “To what extent do the following barriers prevent you from prescribing immunosuppressants for patients with AE?”

Among the respondents who claimed they never prescribed immunosuppressants, we investigated the reasons that affected the prescribing of immunosuppressants (Fig. 7). The most common strong barriers for the prescription of immunosuppressants were “Patients’ refusal” (78.7%) and “Economic burden on the patient” (77.8%). Lower percentages of respondents identified “Lack of access to such treatment” (69.4%), “Adverse effects” (65.7%), “Insufficient knowledge” (62.0%), “Off-label use” (62.0%), “Lack of access to AE guidelines” (60.2%), and “Poor response” (53.7%) as strong barriers.

### Other practices and related perspectives in the clinical management of patients with AE

Description of the treatment practices (such as choice of initial IT, further treatment choice for patients who respond poorly to first-line IT and timing of ASMs withdrawal), the attitudes regarding the initial IT treatments and the perspectives on factors affecting the prognosis of AE patients can be seen in the eFigure 1–4 and eResults in the supplement.

We investigated the respondents’ preferences of the initial IT (eFigure 1 A in the supplement). Our data suggests 42.0% of the respondents preferred IVMP, 38.6% preferred IVMP combined with IVIG and 15.5% preferred IVIG, which indicated that there is difference in initial IT option between respondents. Then we investigated the reasons that affected respondents’ decision, respondents were asked to answer this question based on their previous clinical experience (eFigure 1B in the supplement). The most common reasons that affected respondents’ decision were “Patients’ economic condition” (75.0%) and “Effectiveness of drugs” (75.0%).

For patients with poor response to the initial IT, the consensus recommends reinitiating first-line IT or initiating immunosuppressants [2, 3]. Therefore, we investigated the prescription preferences of respondents for such patients (eFigure2 in the supplement). Our data shows that the 58.0% of the respondents preferred reinitiating the first-line IT regimens different from initial IT, 13.0% reinitiating the first-line IT regimen same as initial IT, while only 15.5% of them choosing immunosuppressants, which indicated that the rate of prescribing immunosuppressants for such patients among respondents is extremely low.

Patients with AE manifested by seizures commonly, the consensus recommends the use of ASMs for such patients [2, 3]. However, existing consensus does not recommend when the ASMs should be discontinued. Thus, we surveyed how long our respondents prefer to wait until stopping ASMs after IT for AE patient (eFigure3 in the supplement). Our research finds an enormous differences in the time to withdraw ASMs among respondents: from 6 to 12 months (27.5%) to proceed 24 months (15.9%).

Finally, we investigated what factors they believed to affect the prognosis of AE patients, respondents were asked to answer this question based on their previous clinical experience (eFigure4 in the supplement). The most impactful factors they believed were “Inability to complete treatment due to patient’s economic burden” (75.8%) and “Insufficient knowledge about AE” (69.1%).

## Discussion

As far as we know, this survey is the first assessment of AE knowledge, practices and perspectives among neurologists in western China. We found substantial gaps in AE knowledge and some barriers to AE treatment among Chinese neurologist. These insights may help guide public health policies and medical training around AE.

Although our survey showed that neurologists in western part of China, including those who serve as encephalitis experts on the NICG, still lack AE knowledge. In fact, the rate of correct responses to questions about AE knowledge in China was higher among our respondents (68.3%) than the rate of correct responses to questions about other neurological diseases, such as knowledge about Parkinson disease and stroke among neurologists in China (45.8% and 54.9% respectively) [13, 27]. This phenomenon may suggest that AE, an emerging field, has received more attention among Chinese clinical doctors, since the respondents are similar. Our respondents scored particularly poorly on questions related to rare types of AE rarer than anti-NMDAR encephalitis. This phenomenon is in line with expectations and may be similar to the situation in most countries and regions (although there is a lack of data from other regions at present), which may be related to the clinical rarity, late reporting, insufficient publicity and training of these relatively rare AEs. The lack of knowledge and understanding of these may lead to clinical missed diagnosis and treatment, and then cause harm to the health of patients. Take GABA_B_R encephalitis as an example, it is often associated with malignant tumors which can lead to high mortality [28]. Insufficient knowledge about GABA_B_R encephalitis may lead to misdiagnosis of it and other unsuitable clinical practices, such as missed diagnosis of tumor and lose the opportunity of early treatment of tumor. Our research indicated that it is urgent to improve the understanding of rare types of AE in western China.

In western China, there is still part (12.4%) of the neurologists never ordered antibodies tests for patients with suspected AE. Whether there are regional differences in the above phenomena needs further research. We found that failure to order such testing was associated with lower AE knowledge and education level, less senior job title, smaller hospital setting, and lack of membership in the NICG. Therefore, these neurologists should be the key targets for the popularization of AE related knowledge and consensus guidelines.

Although immunosuppressants may improve outcomes in patients who respond poorly to first-line IT [2, 3], over half of our respondents never prescribed immunosuppressants. Another nearly 8% of respondents did not know whether they should prescribe it at all. Our survey on neurologists is consistent with the previous clinical cohort studies of AE in which the application rate of immunosuppressants in Chinese patients is much lower than that in western countries [5, 6, 9, 10]. The reasons lead to such disparity including economic burden, off-label use of medication and potential adverse effects, which have been were speculated by the authors of previous studies on the clinical characteristics and prognosis of AE, including our studies [5, 6, 9, 10], and confirmed by our current survey. In the present study, neurologists who had a higher education level, more AE knowledge or more senior job title or who worked at larger hospitals were more likely to prescribe immunosuppressants and to know whether it should be prescribed be at all. This is consistent with the idea that knowledge is a prerequisite for promoting positive behavior [29]. The results may suggest that improving AE knowledge among clinicians in China may lead to more timely use of immunosuppressants.

In general, the management of AE in western China faces challenges in economy, policy and resource structure. Our results indicate that major barriers to proper AE management include neurologists’ concerns about the economic burden on the patient, off-label use of medication, and potential adverse effects, which have also been supposed by some authors [5, 10]. In this study, the lack of AE antibody detection availability is a prominent factor affecting the diagnosis and treatment of AE in addition to the above reasons. Similarly, lack of access to auxiliary tests or drugs and high treatment costs have also been identified as major barriers to management of other neurological diseases such as epilepsy, Alzheimer’s disease and Parkinson’s disease, whatever in developed or developing countries [16, 30, 31]. Compared with the eastern China, the western China has more complex geographical conditions, relatively fewer medical personnel, and relatively backward infrastructure. Therefore, the lack of health service resources in western China is more common [18]. Further insights into our respondents’ practices and perspectives about initial IT, further treatment of patients who respond poorly to first-line IT, as well as cessation of ASM are discussed in the Supplement.

This survey has several limitations that need to be considered. All neurologists we invited were provided contact information by SMDA and were not randomly selected. The actual gaps on knowledge and practice of AE among neurologists in western China may be underestimated in our study, given that our sample may be biased towards those more knowledgeable and interested in AE. In addition, our results should be interpreted with caution in light of the risk of recall bias, since subjects were asked to respond based on their subjective memory.

## Conclusion

In conclusion, neurologists in western China have understood AE to a certain extent. However, at present, their knowledge in this field is still insufficient. This lack of understanding is related to their education level and the level of the hospital they work in. In western China, the dissemination of AE related medical training and guidance needs to be popularized to individuals with low education level or working in non-academic hospitals. Policies should be formulated to increase the availability of AE related antibody testing or drugs and reduce the economic burden of disease, so as to improve the clinical management of AE patients in western China. The improvement of neurologists’ cognition of AE, the better accessibility of medical resources and the reduction of patients’ medical economic burden will enable more patients with AE to be identified and effectively treated at an early stage, thus saving the lives of a large number of patients and reducing the disease burden of their families.

## Electronic supplementary material

Below is the link to the electronic supplementary material.

Supplementary Material 1

## Data Availability

The datasets used and/or analysed during the current study are available from the corresponding author on reasonable request.

## List of Abbreviations

AE: Autoimmune encephalitis
NMDAR: N-methyl-d-aspartate receptor
LGI1: Leucine-rich glioma-inactivated 1
Caspr2: Contactin-associated protein-like 2
GABA_B_R: γ-aminobutyric acid type B receptor
IVMP: Intravenous methylprednisolone
IVIG: Intravenous immunoglobulin
IT: Immunotherapy
SMDA: Sichuan Medical Doctor Association
NICG: Nervous System Infection and Cerebrospinal Fluid Study Group
ASMs: Anti-seizure medications
KS: Knowledge score
