# Supplementary material for: Limitations on knowledge of autoimmune encephalitis and barriers to its treatment among neurologists: a survey from western China
Source: BMC Neurol. 2023 Mar 7;23:99. doi: 10.1186/s12883-023-03139-0 (PMC9990234; doi:10.1186/s12883-023-03139-0)
Supplement: Supplementary file 1 — Supplementary Material 1 [file 12883_2023_3139_MOESM1_ESM.docx]

**Limitations on knowledge of autoimmune encephalitis and barriers to its treatment among neurologists: a survey from western China**

**I. eMethods**

The questionnaire contained 35 items divided into three parts.

Part A (11 items) requested sociodemographic data (gender, age, education level) and the following data on the respondent’s neurology practice: setting, department, job title, years of experience, number of patients with encephalitis or AE treated per year and membership in the NICG.

Part B (10 items) requested data about the respondent’s AE treatment practices towards specific treatments, such as the situation of ordering of diagnostic antibody testing (possible responses: always, sometimes, never), application of first-line IT or immunosuppressants (second-line IT), and the reasons for their responses. Respondents were also asked about their preferred treatment for patients who respond poorly to first-line IT, and how long they prefer to wait until stopping anti-seizure medications (ASMs) after IT. Respondents were asked what factors they believed to influence prognosis of AE patients.

All those responding to Part B were asked to rate potential barriers or facilitators to AE diagnosis and treatment on a 3-item scale comprising the responses strong, moderate, and weak. Only respondents who reported never ordering diagnostic antibody testing were asked to rate potential barriers to such testing. Analogously, respondents who reported never recommending immunosuppressants were asked to rate potential barriers to that therapy.

Part C (14 items) assessed respondents’ knowledge about AE diagnosis, treatment, and prognosis. Those items covered three disease groups: “all types of AE”, “anti-NMDAR encephalitis”, and “rare types of AE”, which comprised AE involving autoantibodies against GABABR, IgLON5, LGI1, or glial fibrillary acidic protein2. Each item could be answered with true, false, or unsure. A knowledge score (KS) was calculated as the sum of correct responses: when the participants answered correctly, + 1 was added to a sum score, whereas a wrong indication or “Not sure” answer added 0 to the sum score, yielding a total score ranging from 0 to 14 points. KS was dichotomized into “high” (≥ median score in our sample) or “low” (< median score).

**Content validity and test-retest reliability of the knowledge section of the questionnaire**

A pilot study to access the content validity and test-retest reliability of the knowledge section of this questionnaire was conducted. Content validity refers to the extent to which the method of measurement includes the major elements relevant to the concept of interest^1^. Content Validity Inventory (CVI) is commonly used to quantitatively evaluate agreement among experts who review a newly designed tool^1,2^. Our original questionnaire was sent to ten experts who had authored a variety of publications in the AE field and had at least 10 years of related experience. A CVI for the AE questionnaire was developed (eTable 1 in the supplement). On an ordinal scale of 1 to 4, each expert rated the appropriateness of each item as it pertained to AE. Items rated 1 corresponded to not relevant, and a rating of 4 corresponded to extremely relevant. Items rated a 1 or 2 were considered not relevant, and those rated 3 or 4 were considered extremely relevant. Results were then calculated based on item agreement, with 0.80 representing the minimum acceptable outcome^3^. All of 10 experts responded to the CVI of AE knowledge questionnaire. The correlation coefficient for 13 of 14 items was 1.0. One expert rated the question pertaining to anti-GFAP encephalitis as a 2, indicating it is an “interesting issue,” but not as clinically relevant as others. The correlation coefficient for this item was 0.93. The total CVI correlation coefficient was 1.0. Ten neurologists were recruited to participate in the test-retest procedures for the AE knowledge section. On Day One, each participant completed the corresponding questionnaire. Questionnaires were repeated on Day Two (24 hours later)^1^. Participants did not review data pertaining to AE knowledge. Test-retest coefficient correlations were 0.9 for the AE knowledge section.

**II. eResults**

**1.Demographic features of the participants**

The majority (44.1%) of the respondents claimed that they contacted 10-30 patients with all-cause encephalitis each year (member of NICG: 26.5% vs. non-member of NICG: 45.0%, p < 0.05), 22.5% see fewer than 10 (members of the NICG: 2.9% vs. non-members of the NICG: 23.5%, p < 0.05), 17.8% see 31-60 (members of the NICG: 38.2% vs. non-members of the NICG: 16.7%, p < 0.05), 4.5% see more than 90 (members of the NICG: 14.7% vs. non-members of the NICG: 3.9%, p < 0.05), 3.6% see 61-90 (members of the NICG: 11.8% vs. non-members of the NICG: 3.2%, p < 0.05), which shows the proportion of members of the NICG who contacted more patients with all-cause encephalitis per year is higher than that in the non-members of the NICG. Regarding the number of AE cases contacted per year, 42.0% of the respondents stated they contacted 1-5 patients with AE each year, 24.8%, 11.0%, 5.2%, 7.2% of the respondents see 6-10, 11-20, fewer than 1, more than 20 patients with AE, respectively. Similarly, members of the NICG saw more patients with AE per year than non-members of the NICG generally: the proportion of physicians who contacted 11-20 patients per year in members of the NICG was 29.4% (10.0% in non-members of the NICG, p < 0.05), the proportion of physicians which contacted more than 20 patients in members of the NICG was 17.6% (6.8% in non-members of the NICG, p < 0.05).

To summarize, compared to non-members of the NICG, participants in members of the NICG were order and more likely to have a longer time in neurology practice, contacted more encephalitis and AE cases. Participants in members of the NICG were more likely to have higher education level as well as job title. There was no statistical difference among members of the NICG and non-members of the NICG with respect to gender and practice setting (P > 0.05). Respondents who were members of the NICG were older, had a higher education level, more senior job title, had been practicing neurology longer, had contact with more cases of encephalitis and AE annually than non-members.

**2.Practices and perspectives on initial immunotherapy**

Among the respondents, 42.0% of the respondents preferred intravenous methylprednisolone (IVMP) as initial immunotherapy (IT) for the patients diagnosed with AE for the first time, as a similar proportion (38.6%) of respondents preferred intravenous immunoglobulin combined with intravenous methylprednisolone (IPI), 15.5% preferred intravenous immunoglobulin (IVIG), 3.4% claimed that they don’t know about the regimen and 0.5% prefer other (IVMP + plasma exchange) regimen (eFigure1.A in the supplement). Physicians in AE knowledge level and educational level subgroups had significantly different preferences proportion for initial IT. Most of the respondents (45.9%) with KS ≥ 10 preferred IPI, while most of the respondents with KS < 10 preferred IVMP (48.6%, p < 0.001). Most of the respondents (65.4%) with doctorate degree preferred IPI, while most of the respondents with bachelor degree preferred IVMP (44.6%, p < 0.001), and most of the respondents with master degree preferred IVMP (45.8%, p < 0.001). In conclusion, neurologists with highest education level, higher AE knowledge level were more likely to choose IPI rather than IVMP. Comparisons between other subgroups (job title, years practicing neurology, practice setting, membership in NICG) provided no significant difference in the proportion of regimen preferred (p > 0.05).

Perspectives on facilitators determining the initial IT regimen are showed in eFigure1.B in the supplement. When asked about the extent of potential facilitators, 75.0% of the respondents rated strong for the facilitator “Patients' economic condition”, 75.0% of the respondents rated strong for the facilitator “Effectiveness of drugs”. Regarding to the facilitators “Expert consensus recommendations”, “Safety of drugs”, “Availability of drugs” and “Patients' preference”, the proportion of respondents who rated strong was 74.5%, 67.0%, 66.0% and 64.0%, respectively. In short, the most impactful facilitators for respondents were patient's economic condition and efficacy, followed by expert consensus recommendations, safety, availability, patient's preference.

**3.Practices on patients who had a poor response to the first-line immunotherapy**

The majority (58.0%) of the respondents preferred reinitiating the different first-line IT regimens for the patients that had a poor response (2 weeks after treatment) to the first-line IT, while 15.5% choosing immunosuppressants, 13.0% reinitiating the same first-line IT regimen, 4.8% switching to strengthening symptomatic treatment, 5.8% don’t know about regimen, 2.9% transferring the patient to other hospitals (eFigure2 in the supplement). The proportion of preference regimen in subgroups of job title, time in neurology practice, practice setting, member of NICG and education level both provided no significant difference, besides AE knowledge level subgroup. Most of the respondents (63.7%) with KS < 10 preferred reinitiating the different first-line IT regimen, while the proportion for reinitiating the different first-line IT in KS ≥ 10 group was 47.2% (p < 0.05).

**4.Practices on time to stops ASMs**

When asked about the time to stops ASMs (anti-seizure medicines) for patients with AE after the IT, the most (27.5%) of the respondents claimed that they stop ASMs for patients with AE 6 to 12 months after the IT, followed by within 6 months (20.3%), 12 to 24 months (16.4%), proceed 24 months (15.9%), don’t know when to stop (15.0%), others (4.8%) (eFigure3 in the supplement). Respondents with KS < 10 had significantly higher proportion (29.2%) choosing “Don’t know when to stop” than respondents with KS ≥ 10 (7.4%, p < 0.05). There was no statistically significant difference in the proportion of ASMs withdrawal time in other subgroups.

**5.Perspectives on prognosis of AE**

When asked about the extent of the potential factors impact the prognosis of patients with AE, 75.8% of the respondents rated strong for the factor “Economic burden on the patient”, 69.1% of the respondents rated strong for the factor “Insufficient knowledge” (eFigure4 in the supplement). Regarding to the factors “Medical insurance restricts the use of certain drugs”, “Lack of access to such treatment”, “Lack of access to antibody tests” and “Lack of AE guideline”, the proportion of respondents which rated strong was 67.6%, 65.7%, 58.5%, and 56.0%, respectively. In short, the most impactful factor for respondents was the “Inability to complete treatment due to patient’s economic burden”; the next impactful factor was insufficient knowledge about AE, followed by medical insurance restricts the use of certain drugs, lack of access to such treatment, lack of access to antibody tests, lack of access to AE guideline.

**III. eDiscussion**

There is widely recognized that prompt diagnosis and timely first-line IT lead to improvement or full recovery in 70.0-80.0% of the cases^4,5^. For patients with AE, consensus recommends that first-line IT include IVMP, IVIG, and plasma exchange^6^. Our results suggest a favorable clinical status that almost all the respondents (96.1%) use any type of first-line IT as initial IT. However, it remains unstudied which regimen clinicians preferred as initial IT. Our study is timely in providing the first insight into this topic: for the patients diagnosed with AE for the first time, eighty-seven of 207 (42.0%) respondents prefer IVMP as initial IT, as a similar proportion (38.6%) prefer IPI, 15.5% prefer IVIG. Most of the respondents (65.4%) with doctorate degree preferred IPI, while most of the respondents with bachelor degree (44.6%, p < 0.05) and master degree (45.8%, p < 0.05) preferred IVMP. Similarly, most of the respondents (45.9%) with KS ≥ 10 preferred IPI, while most of the respondents with KS < 10 preferred IVMP (48.6%, p < 0.001). The reason why respondents with higher education level or higher AE knowledge level prefer the IPI rather than IVMP is not yet known; whether differences exist in the prognosis of patients with different regimens need more research.

For the patients that had poor response to the first-line IT, consensus recommends initiating immunosuppressants or reinitiating the first-line IT^6^. It remains unstudied which regimen clinicians preferred for patients who had poor response to the initial first-line IT. We investigated it for the time, which shows that the majority (58.0%) of the respondents preferred reinitiating the different first-line IT regimen, whereas only 15.5% of the respondents choosing Immunosuppressants. Regarding the reasons for choosing initial IT, patients' economic condition, efficacy, expert consensus recommendations, safety, availability, patients' preference were both completely or strongly considered by neurologists when making decisions. Moreover, patient's economic burden is acknowledged by most of the respondents which completely or strongly impact the prognosis of AE patients.

The International League Against Epilepsy proposed conceptual definitions for two main diagnostic entities (“acute symptomatic seizures secondary to autoimmune encephalitis” and “autoimmune-associated epilepsy”) in 2020^7^. Given this background, we are interested in the time when neurologists are stopping ASMs for patients with AE after the IT. Our research finds an enormous gap in the time to stop ASMs (from 6-12 months (27.5%) to proceed 24 months (15.9%)) between neurologists, while considerable neurologists don't know when to stop the ASMs (31/207, 15.0%). Respondents with KS < 10 have a significantly higher proportion (21/72, 29.2%) of “Don’t know when to stop” than respondents with KS ≥ 10 (10/135, 7.4%, p < 0.05). There are still insufficient data on whether seizure in patients with AE can develop into chronic epilepsy and when to stop ASMs. The practice gap in stopping ASMs in our study calls for further research about seizure outcomes to guide the use of ASMs for patients with AE.

**References**

1. Long L, McAuley J, Shneker B, Moore J. The validity and reliability of the Knowledge of Women's Issues and Epilepsy (KOWIE) Questionnaires I and II. *The Journal of neuroscience nursing : journal of the American Association of Neuroscience Nurses.* 2005;37(2):88-91.

2. Anders R, Tomai J, Clute R, Olson T. Development of a scientifically valid coordinated care path. *The Journal of nursing administration.* 1997;27(5):45-52.

3. Summers S. Establishing the reliability and validity of a new instrument: pilot testing. *Journal of post anesthesia nursing.* 1993;8(2):124-127.

4. Ropper AH, Dalmau J, Graus F. Antibody-Mediated Encephalitis. *New England Journal of Medicine.* 2018;378(9):840-851.

5. Al M. Treatment and prognostic factors for long-term outcome in patients with anti-NMDA receptor encephalitis: an observational cohort study. 2013.

6. Expert consensus on diagnosis and treatment of autoimmune encephalitis in China. *Chinese Journal of Neurology.* 2017;050(002):91-98.

7. Acute symptomatic seizures secondary to autoimmune encephalitis and autoimmune‐associated epilepsy: Conceptual definitions. *Epilepsia.* 2020.

**IV. eFigures**

**
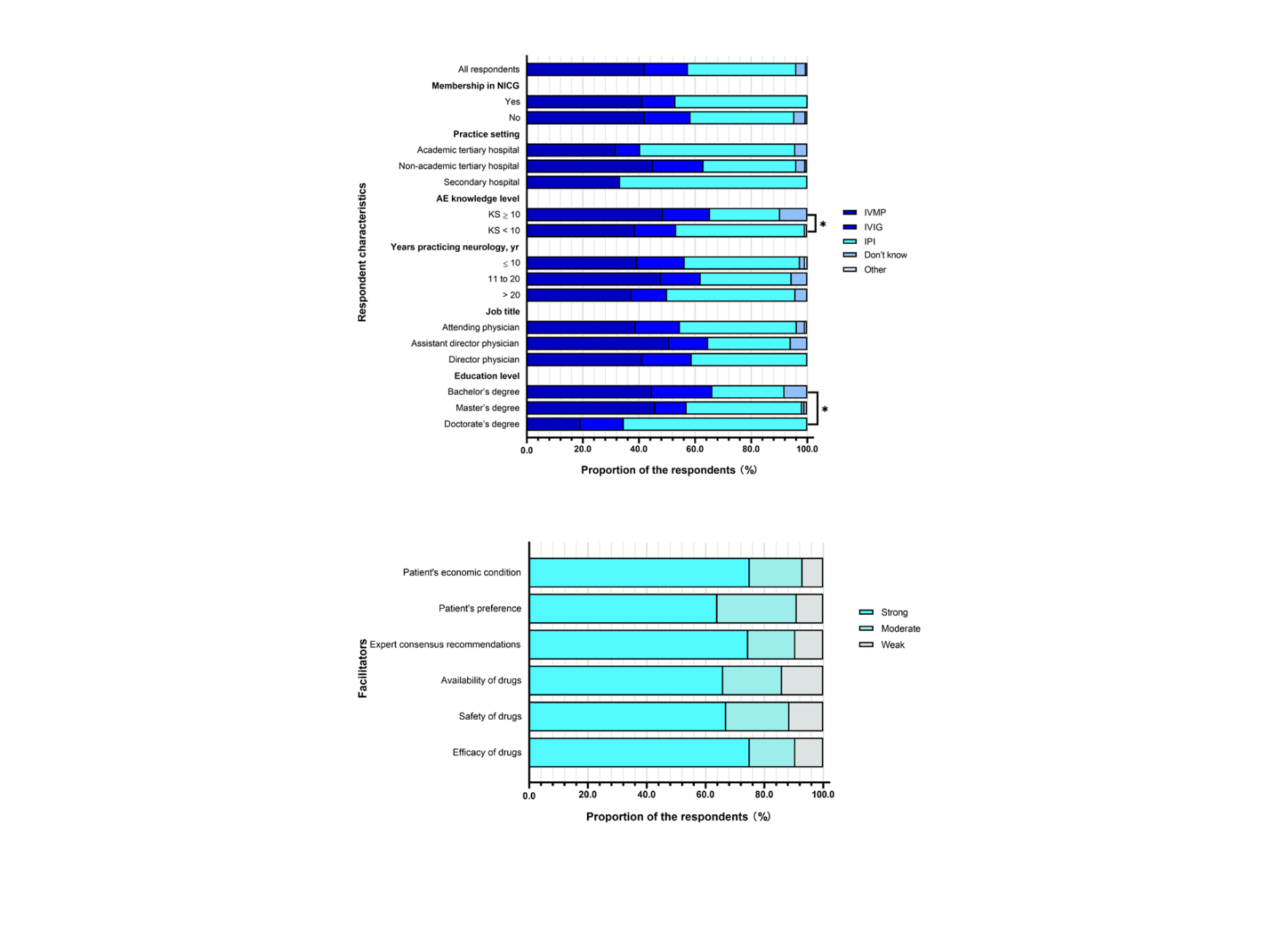
**

**eFigure 1. Practice and perspective on immunotherapy regimen for the patients diagnosed with autoimmune encephalitis for the first time.** (A) Responses to the question “Which IT regimen did you prefer for the patients diagnosed with AE for the first time previously?” * two-sided p < 0.05. (B) Responses to the question “To what extent do any of the following facilitators that impact you choosing IT regimen for the patients diagnosed with AE for the first time previously?”.

Other included: IVMP + plasma exchange

IVIG, intravenous immunoglobulin; IVMP, intravenous methylprednisolone; IPI, intravenous immunoglobulin combined with intravenous methylprednisolone; IT, immunotherapy; AE, autoimmune encephalitis; KS, knowledge scores.


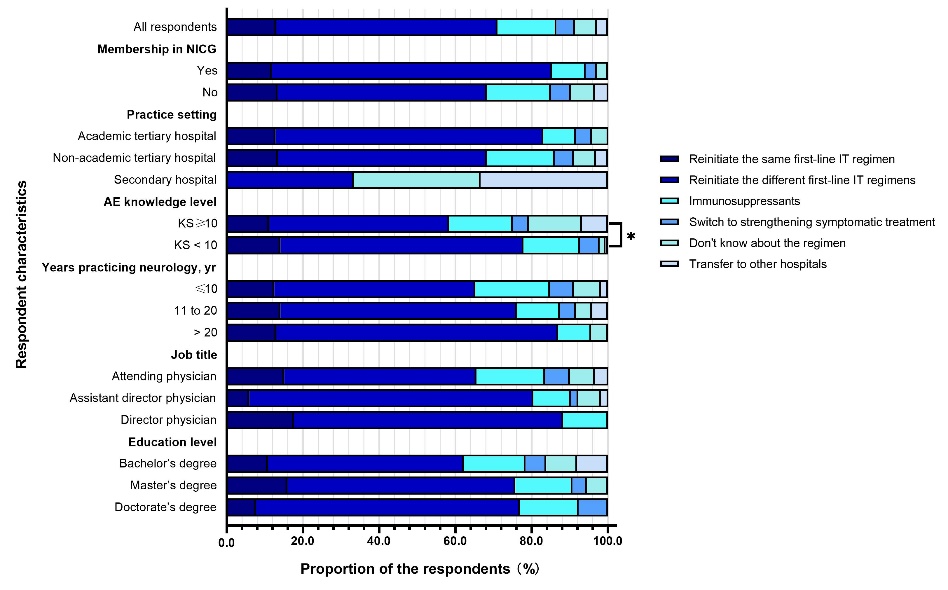


**eFigure 2. Practice on regimen for patients who had poor response to the first-line immunotherapy among neurologists in different characteristic subgroups in western China.** Responses to the question “Which regimen did you prefer for the patients that had a poor response to the first-line IT (2 weeks after treatment) previously?” * two-sided p < 0.05.

IT, immunotherapy; KS, knowledge scores


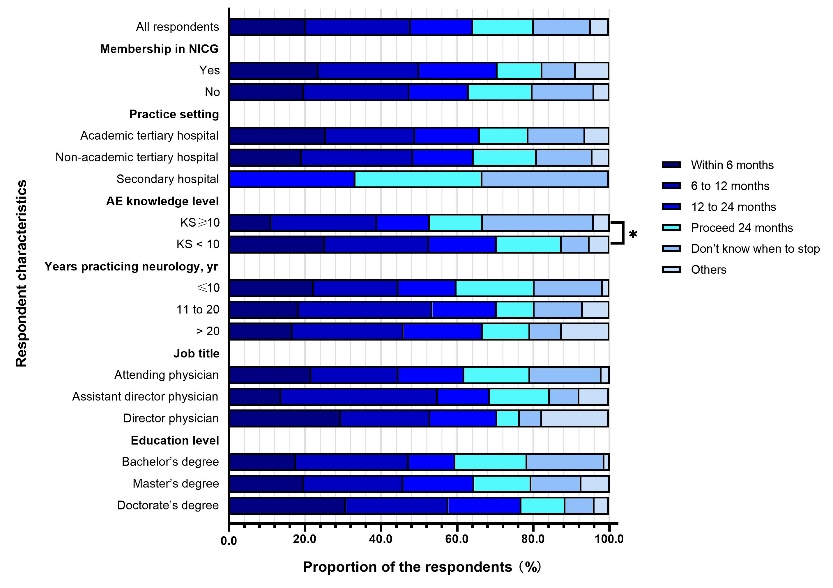


**eFigure 3. Practice on anti-seizure medicines for patients with autoimmune encephalitis after the immunotherapy among neurologists in different characteristic subgroups in western China.** Responses to the question “When did you stop anti-seizure medicines for patients with AE after the IT previously?” * two-sided p < 0.05.

Other included: evaluating whether to discontinue based on seizure outcomes, evaluate whether to discontinue based on seizure outcomes and video electroencephalography.

AE, autoimmune encephalitis; IT, immunotherapy; ASMs, anti-seizure medicines; KS, knowledge scores.


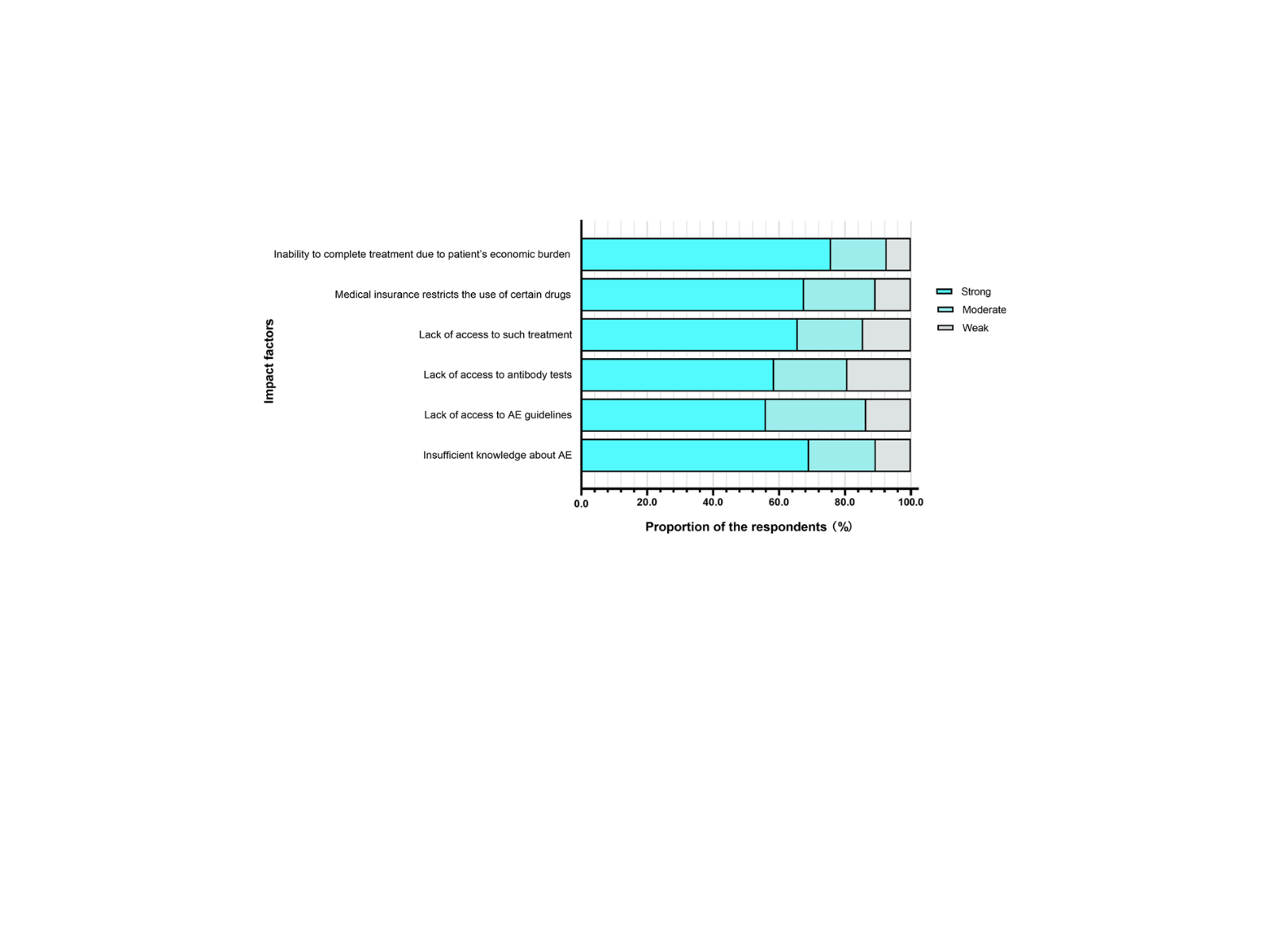


**eFigure 4. Perspective on factors that impact the prognosis of patients with autoimmune encephalitis among neurologists in western China.** Responses to the question “According to your clinical experience, to what extent do any of the following factors that impact the prognosis of patients with AE?”.

AE, autoimmune encephalitis.

**eTable 1. Content Validity Inventory of autoimmune encephalitis knowledge section**

| **On an ordinal scale of one (1) to four (4), with (1) denoting an irrelevant item and (4) denoting an extremely relevant item, please rate the following questions pertaining to its relevance to autoimmune encephalitis knowledge.** | | | | |
| --- | --- | --- | --- | --- |
|  | 1=Not relevant;  4=Highly relevant | | | |
| If a patient is negative for AE-related antibodies, a diagnosis of AE can be ruled out. (F) | 1 | 2 | 3 | 4 |
| Patients with AE usually have an elevated number of nucleated cells in cerebrospinal fluid. (F) | 1 | 2 | 3 | 4 |
| Many patients with AE show obvious brain abnormalities by magnetic resonance imaging. (F) | 1 | 2 | 3 | 4 |
| The main clinical manifestations of anti-NMDAR encephalitis are psychiatric symptoms, seizures, dyskinesia, and disturbed consciousness. (T) | 1 | 2 | 3 | 4 |
| Some patients with anti-NMDAR encephalitis show abnormal delta brushes in electroencephalography. (T) | 1 | 2 | 3 | 4 |
| The most frequent tumor among patients with anti-GABA_B_R encephalitis is teratoma. (F) | 1 | 2 | 3 | 4 |
| The main clinical manifestations of anti-IgLON5 encephalitis are parasomnia, sleep disorder, cognitive impairment, and gait abnormality. (T) | 1 | 2 | 3 | 4 |
| Brain magnetic resonance imaging of some patients with anti-GFAP encephalitis shows linear perivascular enhancement that extends radially from the ventricles. (T) | 1 | 2 | 3 | 4 |
| Immunotherapy is the core treatment for patients with AE. (T) | 1 | 2 | 3 | 4 |
| Active tumor removal is recommended for patients with both anti-NMDAR encephalitis and teratoma who develop disturbed consciousness. (T) | 1 | 2 | 3 | 4 |
| AE usually shows a single course and does not recur. (F) | 1 | 2 | 3 | 4 |
| Most patients with anti-NMDAR encephalitis have good long-term functional outcomes. (T) | 1 | 2 | 3 | 4 |
| More than 80% of patients with GABA_B_R encephalitis have good long-term prognosis and extremely low risk of mortality. (F) | 1 | 2 | 3 | 4 |
| Patients with anti-LGI1 encephalitis are more likely to show cognitive dysfunction than patients with other types of AE. (T) | 1 | 2 | 3 | 4 |

Abbreviations: AE = autoimmune encephalitis; EEG = electroencephalography; MRI = magnetic resonance imaging; F = false; T = Ture; NMDAR =anti-N-methyl-D-aspartate receptor; GABA_B_R = anti-γ-aminobutyric acid receptor type B; LGI1 = anti-leucine-rich glioma-inactivated 1; GFAP = anti-glial fibrillary acidic protein.

**eAppendix. Survey on the knowledge, practices and perspective on autoimmune encephalitis**

**(The English version of the questionnaire)**

You are being asked to participate in a research survey that may help us to understand the knowledge, practices and perspectives on neurologists in autoimmune encephalitis. You are being contacted to participate in this research survey because you are involved

in the clinical practice of neurology. Your participation is voluntary. The survey is anonymous. All data will be stored in a secure drive. Data will only be accessible to the study team. There is no personal benefit from your participation in the survey. It will take you 5-8 minutes in total. Thank you very much for your cooperation.

The following questions are single-choice unless otherwise specified.

**Part A. participant’s characteristics.**

Q1: You are:

Male

Female

Q2: Your age is (years):

< 22

22-30

31-45

46-65

> 65

Q3: The setting you currently practice (please specify):

Q4: The level of your currently practice setting:

Academic tertiary hospital

Non-academic tertiary hospital

Secondary hospital

Primary hospital

Q5: Whether practiced as neurologists in neurology department currently?

Yes

No

Q6: Time in neurology practice (years):

≤ 10

11-20

> 20

Q7: Your job title is:

House physician

Attending physician

Assistant director physician

Director physician

Q8: Your education level is:

Below bachelor

Bachelor

Master

Doctorate

Q9: How many all-cause encephalitis cases which are contacted per year?

< 10

10-30

31-60

61-90

> 90

Not sure

Q10: How many autoimmune encephalitis cases which are contacted per year?

< 1

1-5

6-10

11-20

> 20

Not sure

Q11: Are you a member of Neurological system infection and cerebrospinal fluid Study Group of Sichuan Medical Association (NICG)?

Yes

No

**Part B. practices and perspectives**

Q12: How often have you ordered diagnostic antibody testing for patients with suspected AE?

Always

Sometimes

Never

Q13. To what extent do the following barriers prevent you from ordering diagnostic antibody testing for patients with suspected autoimmune encephalitis?

|  | Strong | Moderate | Weak |
| --- | --- | --- | --- |
| Lack of access to AE guidelines |  |  |  |
| Insufficient knowledge |  |  |  |
| Lack of access to antibody tests |  |  |  |
| Patient's preference |  |  |  |
| Economic burden on the patient |  |  |  |

Q14. Which IT regimen did you prefer for the patients diagnosed with AE for the first time previously?

Intravenous methylprednisolone

Intravenous immunoglobulin

Plasma exchange

Intravenous immunoglobulin combined with intravenous methylprednisolone

Immunosuppressant

Don’t use immunotherapy

Don’t know about the regimen

Other (please specify):

Q15. To what extent do any of the following facilitators that impact you choosing IT regimen for the patients diagnosed with AE for the first time previously?

|  | Strong | Moderate | Weak |
| --- | --- | --- | --- |
| Efficacy of drugs |  |  |  |
| Safety of drugs |  |  |  |
| Availability of drugs |  |  |  |
| Expert consensus recommendations |  |  |  |
| Patient's preference |  |  |  |
| Patient's economic condition |  |  |  |

Q16. To what extent do any of the following barriers that keep you from prescribing IT for the patients diagnosed with AE for the first time previously?

|  | Strong | Moderate | Weak |
| --- | --- | --- | --- |
| Economic burden on the patient |  |  |  |
| Insufficient knowledge |  |  |  |
| Lack of access to such treatment |  |  |  |
| Patient's preference |  |  |  |
| Lack of access to AE guidelines |  |  |  |
| Poor response |  |  |  |
| Adverse effect |  |  |  |
| Off-label use |  |  |  |

Q17. Which regimen did you prefer for the patients that had a poor response to the first-line IT (2 weeks after treatment) previously?

Reinitiate the same first-line immunotherapy regimen

Reinitiate the different first-line immunotherapy regimens

Immunosuppressants

Switch to strengthening symptomatic treatment

Don't know about the regimen

Transfer to other hospitals

Other (please specify):

Q18. Do you ever prescribe immunosuppressants for the patients with AE?

Yes

No

Don’ know whether should use.

Q19. To what extent do any of the following barriers that keep you from prescribing immunosuppressants?

|  | Strong | Moderate | Weak |
| --- | --- | --- | --- |
| Lack of access to AE guidelines |  |  |  |
| Insufficient knowledge |  |  |  |
| Lack of access to such treatment |  |  |  |
| Patient's refusal |  |  |  |
| Economic burden on the patient |  |  |  |
| Poor response |  |  |  |
| Adverse effect |  |  |  |
| Off-label use |  |  |  |

Q20. When did you stop anti-seizure medicines for patients with AE after the immunotherapy previously?

Within 6 months

6 to 12 months

12 to 24 months

Proceed 24 months

Don’t know when to stop

Other (please specify):

Q21. According to your clinical experience, to what extent do any of the following factors that impact the prognosis of patients with AE?

|  | Strong | Moderate | Weak |
| --- | --- | --- | --- |
| Insufficient knowledge about AE |  |  |  |
| Lack of access to AE guidelines |  |  |  |
| Lack of access to antibody tests |  |  |  |
| Lack of access to such treatment |  |  |  |
| Medical insurance restricts the use of certain drugs |  |  |  |
| Inability to complete treatment due to patient’s economic burden |  |  |  |

**Part C. knowledge.**

Q22. If a patient is negative for AE-related antibodies, a diagnosis of AE can be ruled out.

True

False

Not sure

Q23. Patients with AE usually have an elevated number of nucleated cells in cerebrospinal fluid.

True

False

Not sure

Q24. Many patients with AE show obvious brain abnormalities by magnetic resonance imaging.

True

False

Not sure

Q25. The main clinical manifestations of anti-NMDAR encephalitis are psychiatric symptoms, seizures, dyskinesia, and disturbed consciousness.

True

False

Not sure

Q26. Some patients with anti-NMDAR encephalitis show abnormal delta brushes in electroencephalography.

True

False

Not sure

Q27. The most frequent tumor among patients with anti-GABA_B_R encephalitis is teratoma.

True

False

Not sure

Q28. The main clinical manifestations of anti-IgLON5 encephalitis are parasomnia, sleep disorder, cognitive impairment, and gait abnormality.

True

False

Not sure

Q29. Brain magnetic resonance imaging of some patients with anti-GFAP encephalitis shows linear perivascular enhancement that extends radially from the ventricles.

True

False

Not sure

Q30. Immunotherapy is the core treatment for patients with AE.

True

False

Not sure

Q31. Active tumor removal is recommended for patients with both anti-NMDAR encephalitis and teratoma who develop disturbed consciousness.

True

False

Not sure

Q32. AE usually shows a single course and does not recur.

True

False

Not sure

Q33. Most patients with anti-NMDAR encephalitis have good long-term functional outcomes.

True

False

Not sure

Q34. More than 80% of patients with GABA_B_R encephalitis have good long-term prognosis and extremely low risk of mortality.

True

False

Not sure

Q35. Patients with anti-LGI1 encephalitis are more likely to show cognitive dysfunction than patients with other types of AE.

True

False

Not sure
